# Real-world effects and adverse events of romosozumab in Japanese osteoporotic patients: A prospective cohort study

**DOI:** 10.1016/j.bonr.2021.101068

**Published:** 2021-04-16

**Authors:** Tomonori Kobayakawa, Takako Suzuki, Masaki Nakano, Makoto Saito, Akiko Miyazaki, Jun Takahashi, Yukio Nakamura

**Affiliations:** a Kobayakawa Orthopedic and Rheumatologic Clinic, 1969 Kunou, Fukuroi, Shizuoka 437-0061, Japan; b Department of Orthopaedic Surgery, Shinshu University School of Medicine, 3-1-1 Asahi, Matsumoto, Nagano 390-8621, Japan; c Department of Human Nutrition, Faculty of Human Nutrition, Tokyo Kasei Gakuin University, 22 Sanban-cho, Chiyoda-ku, Tokyo 102-8341, Japan; d Department of Clinical Support Office, Tokyo Metropolitan Cancer and Infectious Diseases Center Komagome Hospital, 3-18-22 Honkagome, Bunkyou-ku, Tokyo 113-8677, Japan

**Keywords:** Adverse event, Bone mineral density, Bone turnover marker, Osteoporosis, Romosozumab

## Abstract

Real-world data on the new anti-sclerostin antibody drug, romosozumab, remain scarce. There is a strong need to accumulate and analyze data on romosozumab treatment for such conditions as osteoporosis. The purpose of this study was to investigate the therapeutic and adverse effects of romosozumab for osteoporosis treatment in clinical practice. Of the 230 osteoporosis patients prescribed romosozumab from September 2019 in this prospective multicenter cohort study, 204 patients completed 12 months of treatment. The primary outcome of interest was the rate of change in bone mineral density (BMD) of the lumbar spine, total hip, and femoral neck as measured by dual-energy X-ray absorptiometry. Secondary outcomes included changes in bone turnover markers and serum-corrected calcium level as well as the incidence of adverse events. At 6 and 12 months of romosozumab treatment, the respective percentage change in BMD from baseline was 7.4% and 12.2% for the lumbar spine, 1.8% and 5.8% for the total hip, and 2.9% and 6.0% for the femoral neck, all of which were significantly higher (*P* < 0.001) than baseline values. Patients who switched from another osteoporosis regimen exhibited significantly lower lumbar spine BMD gains versus treatment-naïve patients, especially for cases switching from denosumab. P1NP was significantly increased at 6 months (58.9%; *P* < 0.01), while TRACP-5b was significantly decreased at 6 months (−14.7%; *P* < 0.001) and 12 months (−18.8%; *P* < 0.001) versus baseline values. The largest rate of decrease in serum-corrected calcium was 3.7% at 12 months. Sixty-four (27.8%) of 230 patients experienced an adverse event, and 7 (3.0%) new fractures were recorded. In sum, romosozumab treatment for 12 months significantly improved lumbar spine, total hip, and femoral neck BMD according to real-world data.

## Highlights

• Romosozumab for 12 months significantly improved BMD according to real-world data.
• Switching from denosumab and bisphosphonate displayed lower gains in lumbar BMD.
• The co-administration of a vitamin D3 analogue might prevent hypocalcemia.

## 1 Introduction

Besides bisphosphonates, denosumab and teriparatide are also widely used for patients with osteoporosis. They are undoubtedly effective treatments (Cummings et al., 2009; McClung et al., 2005; Leder et al., 2015) but carry several disadvantages: denosumab is associated with multiple compression fractures after its discontinuation (Cummings et al., 2018). Similarly, teriparatide is reported to be an effective therapeutic drug to reduce the risk of hip fractures (Diez-Perez et al., 2019), however, according to the Guidelines for Prevention and Treatment of Osteoporosis (2011) in Japan, the clinical evidence of the efficacy of teriparatide on hip fracture prevention is still insufficient, and it is ranked as grade C (Orimo et al., 2012) regarding the evaluation grading in the guideline. Some patients also consider the strict daily drug administration of teriparatide inconvenient (Leder et al., 2015). In consultations with those patients, it became apparent to improve BMD as soon as possible to prevent further fractures and maintain patient care.

In March 2019, the anti-sclerostin antibody drug romosozumab first became available in clinical practice in Japan for osteoporosis for high-risk fracture cases of osteoporosis (Assessment of Fracture Risk and Its Application to screening for Postmenopausal Osteoporosis, 1994; Soen et al., 2013). Romosozumab has the dual effect of promoting bone formation and decreasing bone resorption by inhibiting the suppression of Wnt signaling (Ominsky et al., 2015; Taylor et al., 2016). However, there is little evidence on its therapeutic efficacy and adverse events in the real world due to its short history.

The viewpoint of clinical trials differs considerably from that of analyses of real-world data. Clinical trials are performed to assess drug efficacy and safety in strictly targeted populations. Thus, even if a clinical trial determines a new drug to be effective and safe, whether the drug is efficacious for each patient in the population and its risk-benefit relationship cannot be completely ascertained. The aim of studies on actual medical practice focuses on individual patients encountered by physicians. Accordingly, the purpose of this cohort investigation was to examine the real-world clinical and adverse effects of romosozumab for osteoporosis treatment.

## 2 Materials and methods

### 2.1 Study design and participants

From March 2019, this prospective observational study was conducted at our clinic and 4 affiliated institutions. The subjects were primary and secondary osteoporosis patients who were administered romosozumab for 12 months. All patients were injected subcutaneously with 210 mg of romosozumab at the study onset and then monthly thereafter. Patients with lower 25OHD values were offered an active vitamin D3 analogue and, if not inclined, were recommended to take commercially available vitamin D3 and calcium supplements.

Romosozumab was positively used to treat patients diagnosed as having severe osteoporosis, especially those with a high risk of fracture, regardless of the presence or absence of prior treatment for osteoporosis. The definition of severe osteoporosis by the World Health Organization is a BMD value that is 2.5 standard deviation (SD) or more below the average value for young healthy women (i.e., T-score < −2.5 SD) in the presence of 1 or more fragility fractures, or if BMD at the lumbar spine is less than −3.3 SD, or with 2 or more existing vertebral fractures (Assessment of Fracture Risk and Its Application to screening for Postmenopausal Osteoporosis, 1994; Soen et al., 2013; Marcus et al., 2003). In the present study, patients who fulfilled the criteria of severe osteoporosis were recruited as subjects. The occurrence of fracture did not have to be recent, although the age of fracture occurrence was designated as 45 years or older. Fragility fractures of the skull, facial bones, metacarpals, fingers and toes, pathologic fractures, and fractures associated with severe trauma were excluded. Also, patients were excluded if they had experienced a cardiovascular event within the previous year or exhibited hypocalcemia.

The protocol of this study was approved by the ethics committee of our institution (approval numbers: 4349 and 4351). Written informed consent was obtained from all participants prior to enrollment. This study was conducted following the tenets outlined in the Declaration of Helsinki.

### 2.2 Primary and secondary outcomes of interest

To evaluate the effects of 12-month romosozumab therapy on BMD as the primary outcome of interest, dual-energy X-ray absorptiometry (DXA) was employed using a Prodigy Fuga (GE Healthcare, Madison, WI, USA). The minimum significant change for this model was 2% (Shepherd et al., 2006). Lumbar vertebra DXA measured the lumbar 2–4 levels and excluded any vertebral body with a T-score of 1.0 higher than the vertebral body with the lowest T-score. DXA readings were taken at baseline and at 6 and 12 months of treatment. Primary osteoporosis and secondary osteoporosis were distinguished from interviews and medication history. For a more detailed analysis, subjects were divided into groups with and without a history of osteoporosis treatment to examine the therapeutic effects of romosozumab. Among the subjects with an osteoporosis treatment history, the effects of romosozumab were further evaluated according to the type of pretreatment drug (bisphosphonate, denosumab, or teriparatide).

As secondary outcomes of interest, the serum bone turnover markers of procollagen type 1 N-terminal propeptide 1 (P1NP) and tartrate-resistant acid phosphatase isoform 5b (TRACP-5b) were measured by the enzyme immunoassay (EIA) and chemiluminescent enzyme immunoassay (CLEIA) methods for each subject at baseline and after 6 months and 12 months. A previous report demonstrated that TRACP-5b levels were useful bone resorption markers that demonstrated higher clinical sensitivity and signal-to-noise ratio as compared with serum CTX levels (Nenonen et al., 2005). Serum calcium measurements had an intra-assay coefficient of variation of <4.0% (Ca-AL Type C kit; Serotech, Chitose, Japan). Serum-corrected calcium values were determined and used in the present study (Payne et al., 1973). Measurements of serum-corrected calcium were performed at baseline and at 2 weeks, 4 weeks, 6 months, and 12 months of treatment. The changes in serum-corrected calcium were also examined according to estimated glomerular filtration rate (eGFR) in mL/min/1.73 m^2^, which was designated as ≧ 90 in the normal group, 60 ≦ eGFR < 89 in the mild dysfunction group, 30 ≦ eGFR < 59 in the moderate dysfunction group, and 29 > eGFR in the severe group (Coresh et al., 2003). The rate of change in serum-corrected calcium was also investigated with respect to the absence or presence of a vitamin D3 analogue, such as alfacalcidol or eldecalcitol. Lastly, the incidence of adverse events was recorded throughout the study period.

### 2.3 Statistical analysis

Patient background parameters are expressed as the mean ± SD. P1NP and TRACP-5b are expressed as the median. The changes in percentage or value from baseline to the study time points for BMD, P1NP, and TRACP-5b, as well as changes in percentage for serum-corrected calcium levels, were assessed using the Wilcoxon signed-rank test. The Wilcoxon rank-sum test was employed to evaluate the differences between each group with regards to the percentage changes from baseline for BMD, bone turnover markers, and serum-corrected calcium. Differences between study groups were determined by ANOVA or Fisher's exact test. A Kaplan–Meier curve was constructed to delineate the cumulative incidence of discontinuation during the observation period. A two-tailed *P*-value of <0.05 was considered statistically significant for all analyses. All statistical testing was conducted using R version 3.6.0 (R Core Team, 2019; http://www.R-project.org/).

## 3 Results

### 3.1 Characteristics of patients

**Fig. 1:**
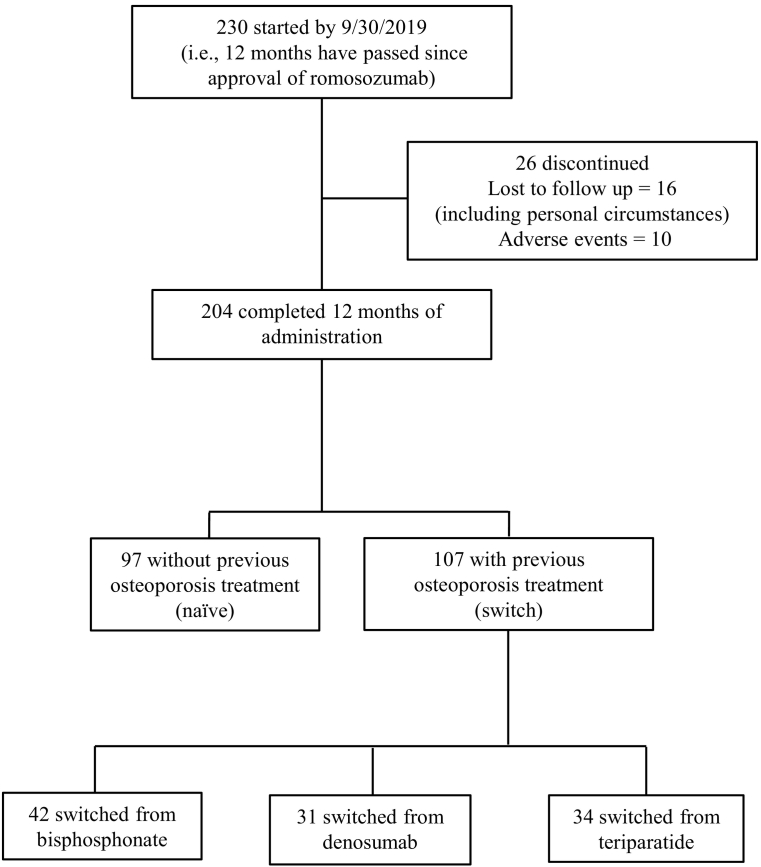
Subject flow diagram throughout the 12-month romosozumab treatment period. Subjects receiving selective estrogen receptor modulators or vitamin D3 as previous treatments were excluded from the study.

A total of 230 subjects were initially enrolled. During the study period, 26 subjects discontinued romosozumab treatment who were either lost to follow-up, citing such personal circumstances as economic reasons (16 subjects), or withdrew due to adverse events (10 subjects). The remaining 204 patients were used in subsequent analyses (Fig. 1). Discontinuation data were excluded from evaluations of romosozumab efficacy.

The baseline demographics and characteristics of the subjects are shown in Table 1. The mean ± SD age was 73.6 ± 13.1 years in this predominantly female (85.3%) cohort. Mean BMD T-scores were −2.56 ± 1.24 for the lumbar spine, −2.51 ± 0.89 for the total hip, and −3.01 ± 0.96 for the femoral neck. Seventy-one (35.1%) subjects had a prevalent vertebral fracture and 52 (25.7%) patients had a history of previous non-vertebral fracture. Over half of the patients (107; 52.5%) had a history of osteoporosis treatment. Among them, 42 (20.6%) patients received bisphosphonates, 31 (15.2%) received denosumab, and 34 (16.7%) received teriparatide. S-Table 1 summarizes the patient characteristics based on primary and secondary osteoporosis. Secondary osteoporosis was defined as diminished bone mass in the presence of some factors, such as an underlying disease or medication. Although glucocorticoids were a major distinguishing factor of secondary osteoporosis in this study involving 35 patients (43%), other factors included rheumatoid arthritis (29 patients; 35.8%), systemic lupus erythematosus (4 patients; 4.9%), malignant tumor/lymphoma (21 patients; 25.9%), diabetes mellitus (7 patients; 8.6%), chronic kidney disease (6 patients; 7.4%), and others (15 patients; 18.5%). The number of patients for each factor was counted redundantly.

**Table 1:** Demographic and Clinical Characteristics of Subjects at Baseline. Data are expressed as the mean ± standard deviation or the number (%) of all patients who completed 12 months of romosozumab treatment. P1NP and TRACP-5b are expressed as median values.

	*N* = 204
Age (years)	73.6 ± 13.1
Female, n (%)	174 (85.3)
Body mass index (kg/m^2^)^a^	21.0 ± 3.3
Bone mineral density T-score	
Lumbar spine	−2.56 ± 1.24
Total hips	−2.51 ± 0.89
Femoral neck	−3.01 ± 0.96
Duration of previous treatment (M)	28
Prior vertebral fracture, n (%)	71 (35.1)
Prior non-vertebral fracture at >45 yrs. of age, n (%)	52 (25.7)
Prior fractures of both vertebrae and non-vertebrae, n (%)	16 (7.9)
Prior osteoporosis treatment, n (%)	107 (52.5)
Bisphosphonate	42 (20.6)
Denosumab	31 (15.2)
Teriparatide	34 (16.7)
Concomitant use of active vitamin D, n (%)	108 (52.9)
Glucocorticoid use, n (%)	35 (17.2)
Median serum total P1NP (IQR), μg/L	64.2 (9.5–495)
Median serum TRACP-5b (IQR), mU/dL	457.5 (81–1500)
Serum albumin, g/dL	4.09 ± 0.35
Serum-corrected calcium, mg/dL	9.28 ± 0.44
Serum phosphorus, mg/dL	3.60 ± 0.56
eGFR, mL/min/1.73 m^2^	67.2 ± 23.5
25OHD, ng/mL	16.0 ± 6.48
ucOC, ng/mL	7.95 ± 7.76
Intact PTH, μg/mL	52.2 ± 42.2

P1NP, procollagen type 1 N-terminal propeptide; IQR, inter-quartile range; TRACP-5b, tartrate-resistant acid phosphatase isoform 5b; eGFR, estimated glomerular filtration rate; 25OHD, 25-hydroxyvitamin D; ucOC, undercarboxylated osteocalcin; intact PTH, intact parathyroid hormone.

a Calculated as weight in kilograms divided by the square of height in meters.

### 3.2 Primary outcomes

**Fig. 2:**
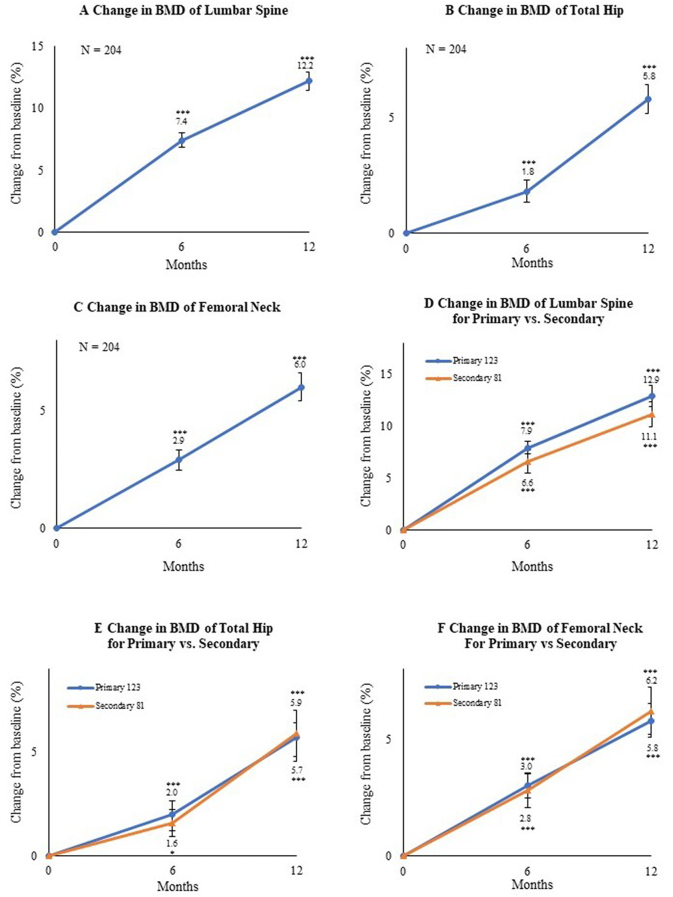
Mean percentage change from baseline to 6 and 12 months in bone mineral density (BMD) of the (A) lumbar spine, (B) total hip, and (C) femoral neck. *P < 0.05, **P < 0.01, and ***P < 0.001 versus baseline (Wilcoxon's signed-rank test). Bars indicate the mean ± standard errors. Mean percentage change in the primary and secondary osteoporosis groups from baseline to 6 months and 12 months in BMD of the (D) lumbar spine, (E) total hip, and (F) femoral neck. Bars indicate the mean ± standard errors. *P < 0.05, **P < 0.01, and ***P < 0.001 versus baseline (Wilcoxon's signed-rank test).

At 6 and 12 months of romosozumab treatment, the percentage change in BMD from baseline was 7.4% and 12.2% for the lumbar spine, 1.8% and 5.8% for the total hip, and 2.9% and 6.0% for the femoral neck in overall subjects. All increases were significant (*P* < 0.001) versus baseline values (Fig. 2A-C).

We also investigated BMD at 6 months and 12 months of romosozumab treatment based on the presence of primary or secondary osteoporosis. The percentage change in BMD from baseline for primary osteoporosis was 7.9% (*P* < 0.001) and 12.9% (*P* < 0.001) for the lumbar spine, 2.0% (*P* < 0.001) and 5.9% (*P* < 0.001) for the total hip, and 3.0% (*P* < 0.001) and 6.2% (*P* < 0.001) for the femoral neck, while that for secondary osteoporosis was 6.6% (*P* < 0.001) and 11.1% (*P* < 0.001) for the lumbar spine, 1.6% (*P* < 0.05) and 5.7 (*P* < 0.001) for the total hip, and 2.8% (*P* < 0.001) and 5.8% (*P* < 0.001) for the femoral neck. There were no significant differences between the primary and secondary groups at any time point (Fig. 2D-F).

**Fig. 3:**
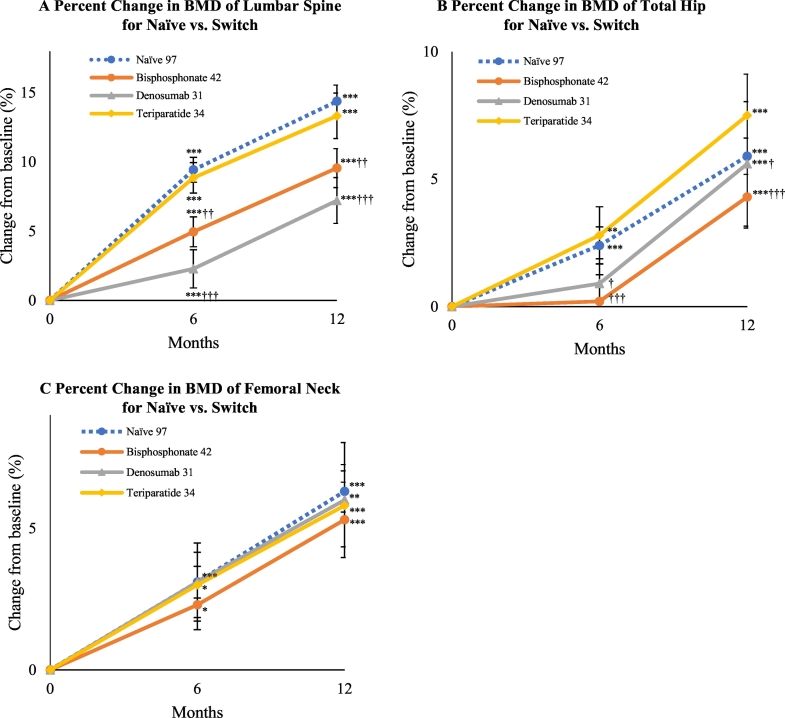
Mean percentage change in bone mineral density (BMD) of the (A) lumbar spine, (B) total hip, and (C) femoral neck from baseline to 6 and 12 months depending on previous therapy. Bars indicate the mean ± standard errors. *P < 0.05, **P < 0.01, and ***P < 0.001 versus baseline (Wilcoxon's signed-rank test). †*P* < 0.05, ††*P* < 0.01, and †††*P* < 0.001 versus treatment-naïve group (Wilcoxon's rank-sum test).

Table 2 summarizes the demographic and clinical characteristics of the subjects to examine the efficacy of romosozumab according to previous treatments. Notably, lumbar spine BMD was comparably higher at baseline in patients switching from denosumab. Lumbar spine BMD respectively increased by 9.4% and 14.4% at 6 months and 12 months in the treatment-naïve group, 5.0% and 9.6% in the bisphosphonate group, 2.3% and 7.2% in the denosumab group, and 8.9% and 13.3% in the teriparatide group as compared with baseline values. All increases were significant (*P* < 0.001) versus baseline. We observed a significant difference in lumbar BMD gains for the bisphosphonate group (*P* < 0.01) and denosumab group (*P* < 0.001) as compared with the treatment-naïve group (Fig. 3A). Total hip BMD respectively increased by 2.4% (*P* < 0.001) and 5.9% (*P* < 0.001) at 6 months and 12 months in the treatment-naïve group, 0.2% (*P* = 0.78) and 4.3% (*P* < 0.001) in the bisphosphonate group, 0.9% (*P* = 0.52) and 5.6% (*P* < 0.001) in the denosumab group, and 2.8% (*P* < 0.01) and 7.5% (*P* < 0.001) in the teriparatide group as compared with baseline values (Fig. 3B). There were significant differences for the bisphosphonate group (*P* < 0.001) and denosumab group (*P* < 0.05) as compared with the treatment-naïve group at 6 and 12 months. Femoral neck BMD respectively increased by 3.1% (*P* < 0.001) and 6.3% (*P* < 0.001) at 6 months and 12 months in the treatment-naïve group, 2.3% (*P* < 0.05) and 5.3% (*P* < 0.001) in the bisphosphonate group, 3.1% (*P* = 0.06) and 6.0% (*P* < 0.01) in the denosumab group, and 3.0% (*P* < 0.05) and 5.8% (*P* < 0.001) in the teriparatide group as compared with baseline values. No remarkable differences from the treatment-naïve group were noted (Fig. 3C). As shown in Table 2, patient BMD levels differed among the treatment-naïve group and previously treated groups. In comparisons among the groups, the mean values of BMD at each site (S-Fig. 1A-C) and the values of gained BMD versus baseline (S-Fig. 1D-F) showed comparable findings at 6 months and 12 months of treatment.

**Table 2:** Demographic and Clinical Characteristics of Subject Groups (treatment-naïve group, bisphosphonate group, denosumab group, and teriparatide group) at Baseline. Data are expressed as the mean ± standard deviation or the number (%) of patients. P1NP and TRACP-5b are expressed as median values.

	Naïve *N* = 97	Bisphosphonates *N* = 42	Denosumab *N* = 31	Teriparatide *N* = 34	*P*-value
Age (years)	74.4 ± 13.1	73.4 ± 13.9	69.6 ± 19.1	68.9 ± 16.0	0.407
Female, n (%)	82 (84.5)	36 (85.7)	25 (83.3)	31 (91.2)	0.819
Body mass index (kg/m^2^)^a^	21.3 ± 3.5	20.6 ± 4.6	19.8 ± 4.5	19.5 ± 3.8	0.088
Bone mineral density T-score					
Lumbar spine	−2.85 ± 1.09	−2.39 ± 1.25	−1.70 ± 1.30	−2.78 ± 1.24	*P* < 0.001
Total hip	−2.66 ± 0.84	−2.25 ± 0.98	−2.48 ± 0.88	−2.55 ± 1.03	0.142
Femoral neck	−3.23 ± 0.85	−2.75 ± 1.10	−2.86 ± 0.95	−2.92 ± 1.14	0.145
Duration of previous treatment (M)	–	29	39	16	*P* < 0.001
Prior vertebral fracture, n (%)	35 (36.1)	13 (31.0)	9 (30.0)	15 (44.1)	0.543
Prior non-vertebral fracture at >45 years of age, n (%)	23 (23.6)	10 (23.8)	6 (20.0)	12 (35.3)	0.512
Prior fractures of both vertebrae and non-vertebrae, n (%)	7 (7.2)	2 (4.8)	3 (10.0)	4 (11.8)	0.659
Concomitant active vitamin D, n (%)	51 (52.6)	21 (50.0)	16 (51.6)	20 (58.8)	–
Glucocorticoid use, n (%)	8 (8.2)	8 (19.0)	11 (35.5)	8 (23.5)	0.004
Median serum total P1NP (IQR), μg/L	73.3 (18.0–495)	25.6 (12.2–88.9)	23.6 (9.5–373)	98.9 (39.8–363)	*P* < 0.001
Median serum TRACP-5b (IQR), mU/dL	587.0 (222–1370)	308.5 (121–771)	243.50 (81–1020)	531.0 (262–1500)	*P* < 0.001
Serum albumin, g/dL	4.14 ± 0.38	4.01 ± 0.31	4.08 ± 0.32	4.07 ± 0.30	0.309
Serum-corrected calcium, mg/dL	9.24 ± 0.35	9.38 ± 0.45	9.29 ± 0.53	9.31 ± 0.36	0.303
Serum phosphorus, mg/dL	3.57 ± 0.56	3.45 ± 0.44	3.72 ± 0.65	3.76 ± 0.56	0.097
eGFR, mL/min/1.73 m^2^	67.9 ± 25.6	64.9 ± 17.7	64.3 ± 27.4	70.3 ± 20.1	0.621
25OHD, ng/mL	15.9 ± 6.21	16.7 ± 5.92	18.4 ± 8.81	13.5 ± 5.09	0.017
ucOC, ng/mL	9.10 ± 8.43	2.87 ± 2.31	1.45 ± 1.29	9.50 ± 6.26	*P* < 0.001
Intact PTH, μg/mL	59.1 ± 52.1	43.4 ± 19.9	49.2 ± 44.2	50.4 ± 32.0	0.188

P1NP, procollagen type 1 N-terminal propeptide; IQR, inter-quartile range; TRACP-5b, tartrate-resistant acid phosphatase isoform 5b; eGFR, estimated glomerular filtration rate; 25OHD, 25-hydroxyvitamin D; ucOC, undercarboxylated osteocalcin; intact PTH, intact parathyroid hormone.

Differences between the groups were determined by ANOVA or Fisher's exact test.

a Calculated as weight in kilograms divided by the square of height in meters.

### 3.3 Secondary outcomes

**Fig. 4:**
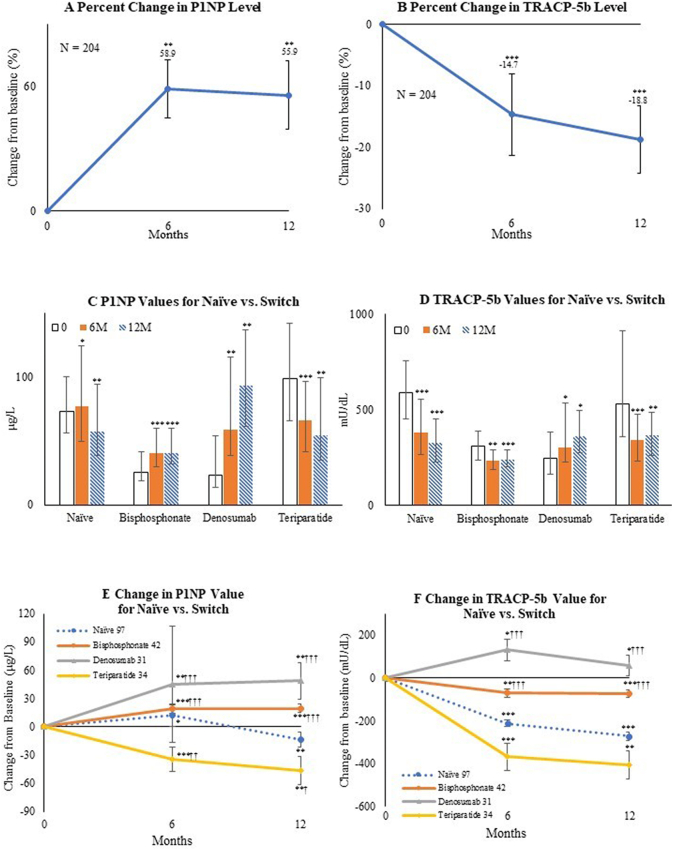
Mean percentage change from baseline to 6 months and 12 months in (A) procollagen type 1 N-terminal propeptide (P1NP) and (B) tartrate-resistant acid phosphatase isoform 5b (TRACP-5b) in overall subjects. Bars indicate the mean ± standard errors. Median value of (C) P1NP (μg/L) and (D) TRACP-5b (mU/dL) depending on the absence or presence of previous treatment at baseline, 6 months, and 12 months. Bars indicate inter-quartile range. Mean value of change from baseline to 6 months and 12 months in (E) P1NP (μg/L) and (F) TRACP-5b (mU/dL) depending on the absence or presence of previous treatment. Bars indicate the mean ± standard errors. *P < 0.05, **P < 0.01, and ***P < 0.001 versus baseline (Wilcoxon's signed-rank test). †*P* < 0.05, ††*P* < 0.01, and †††*P* < 0.001 versus the treatment-naïve group (Wilcoxon's rank-sum test).

The mean changes in P1NP and TRACP-5b levels at 6 months and 12 months from baseline in overall subjects were studied as secondary outcomes. In addition, the median values of serum P1NP and TRACP-5b and their inter-quartile range as well as the mean changes in bone turnover marker values during treatment were compared in the absence or presence of prior treatment. In the cohort, P1NP was significantly increased at 6 months (58.9%; *P* < 0.01) and 12 months (55.9%; *P* < 0.01), while TRACP-5b was significantly decreased at 6 months (−14.7%; *P* < 0.001) and 12 months (−18.8%; *P* < 0.001) versus baseline values (Fig. 4A, B).

In analytical studies by comparisons based on the presence or absence of previous treatment, P1NP was lower in the bisphosphonate and denosumab groups (*P* < 0.001) and TRACP-5b was higher in the treatment-naïve and teriparatide groups (*P* < 0.001) in the clinical characteristics of the patients at baseline (Table 2). Significant changes in both turnover markers during treatment were observed (Fig. 4C-D).

P1NP in the treatment-naïve group was significantly increased (+11.8 μg/L; *P* < 0.05) at 6 months and decreased (−13.5 μg/L; *P* < 0.01) at 12 months as compared with baseline values. P1NP was significantly increased in the bisphosphonate group (6 months: +19.4 μg/L; *P* < 0.001, 12 months: +19.6 μg/L; *P* < 0.001) and denosumab group (6 months: +44.8 μg/L; *P* < 0.01, 12 months: +48.7 μg/L; *P* < 0.01), and significantly decreased in the teriparatide group (6 months: −34.9 μg/L; *P* < 0.001, 12 months: −46.1 μg/L; *P* < 0.01) versus baseline values. There were significant differences among treatment-naïve and switch groups (Fig. 4E).

TRACP-5b was significantly decreased in the treatment-naïve group (6 months: −211.2 mU/dL; *P* < 0.001, 12 months: −270.6 mU/dL; *P* < 0.001), bisphosphonate group (6 months: −70.1 mU/dL; *P* < 0.01, 12 months: −72.4 mU/dL; *P* < 0.001), and teriparatide group (6 months: −368.5 mU/dL; *P* < 0.001, 12 months: −406.7 mU/dL; *P* < 0.01), and significantly increased in the denosumab group (6 months: +131.4 mU/dL; *P* < 0.05, 12 months: +58.3 mU/dL; *P* < 0.05) as compared with baseline values. There were significant differences among the treatment-naïve and switch groups (Fig. 4F).

**Fig. 5:**
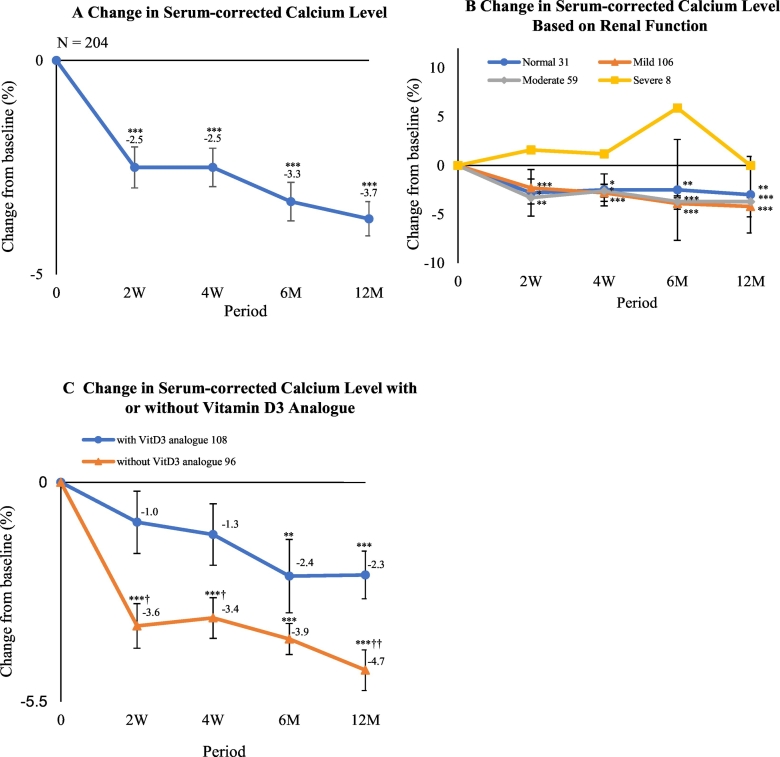
Mean percentage change from baseline to 2 weeks (W), 4 weeks, 6 months (M), and 12 months in serum-corrected calcium level of (A) all subjects, (B) subjects based on renal function, and (C) subjects with or without prescription of a vitamin D analogue. *P < 0.05, **P < 0.01, and ***P < 0.001 versus baseline (Wilcoxon's signed-rank test). †*P* < 0.05, ††*P* < 0.01, and †††*P* < 0.001 versus group with a vitamin D analogue (Wilcoxon's rank-sum test).

The change rate of serum-corrected calcium at 2 weeks of romosozumab treatment was −2.5%, which was significantly different from baseline (*P* < 0.001). The largest rate of change in serum-corrected calcium was −3.7% (*P* < 0.001) at 12 months of romosozumab (Fig. 5A). Regarding renal function, the normal, mild dysfunction, moderate dysfunction, and severe dysfunction groups contained 31, 106, 59, and 8 cases, respectively. There were no remarkable differences in the change rate of serum-corrected calcium according to the degree of renal dysfunction (Fig. 5B). However, the change rate of serum-corrected calcium was significantly lower in subjects with vitamin D3 analogue co-administration (2 weeks: *P* < 0.05, 4 weeks: *P* < 0.05, 12 months: *P* < 0.01 versus subjects without a vitamin D3 analogue) (Fig. 5C). We encountered no cases of clinical hypocalcemia (i.e., CTCAE v5.0 grade 3 or higher, calcium <7.0 mg/dL, and clinical symptoms requiring hospitalization) severe enough to discontinue romosozumab therapy since we recommended patients with a vitamin D deficiency/insufficiency to take active vitamin D preparations. As the administration of active vitamin D carries some risk of hypercalcemia, we did not recommend calcium preparations. For patients not wanting additional medicine, we recommended commercially available supplements of vitamin D or calcium instead.

Kaplan–Meier survival testing was employed to estimate the continuation rate of romosozumab treatment, which was 88.4% during 12 months (S-Fig. 4 in the supplementary materials). A total of 64 adverse events were observed (Table 3). The majority of events were temporary, mild, and did not result in drug discontinuation. The most frequent adverse event was an injection site reaction associated with pain, swelling, and redness lasting 2 days or longer. Ten (4.3%) discontinuation cases due to adverse events were recorded (S-Table2), and 4 (1.7%) serious adverse events occurred. Of note, a cardiovascular event (cerebral infarction) was observed in 1 (0.4%) case during the study period, and 1 (0.4%) case of osteonecrosis of the jaw occurred. The latter patient had been treated with an antiresorptive agent (bisphosphonate preparations) for 6 months prior to romosozumab administration. In addition, 1 (0.4%) patient experienced mild hypocalcemia of grade 2 as defined by the CTCAE v5.0 grading scale during 12 months of treatment without subjective symptoms. The patient did not require drug discontinuation. Seven (3.0%) subjects experienced new fractures during romosozumab treatment. No patient discontinued romosozumab due to a new fracture.

**Table 3:** Adverse Events. Data are expressed as the number of patients (%) and include all patients who received at least 1 romosozumab injection (*N* = 230).

	Total (*N* = 230)
All adverse events	64 (27.8)
Adverse events leading to discontinuation of trial participation	10 (4.3)
Serious adverse events	4 (1.7)
Anterior mediastinal tumor (recurrence of breast cancer)	
Cerebral infarction	1 (0.4)
Osteonecrosis of the jaw	1 (0.4)
Pneumothorax	1 (0.4)
Injection site reaction^a^	32 (13.9)
Pain	18 (7.8)
Swelling	13 (5.7)
Redness	1 (0.4)
Other events of interest	17 (7.4)
Hypozincemia	1 (0.4)
Arrhythmia	1 (0.4)
Fever	2 (0.9)
Anacatesthesia	1 (0.4)
Osteonecrosis of the leg	1 (0.4)
Numbness in limbs	1 (0.4)
Rash	1 (0.4)
Blood pressure elevation	2 (0.9)
Facial flare	1 (0.4)
Fatigue	3 (1.3)
Dysphoria	2 (0.9)
Headache	1 (0.4)
Bloody discharge	1 (0.4)
Hypocalcemia^b^	1 (0.4)
New fractures during romosozumab therapy	7 (3.0)
Thoracic or/and lumbar spine	3 (1.3)
Left proximal tibial fracture	1 (0.4)
Left distal radius fracture	1 (0.4)
Left distal fibular fracture	1 (0.4)
Right proximal humeral fracture	1 (0.4)

a The most frequent adverse event was injection site reaction associated with pain, swelling, and redness lasting 2 days or longer.

b Hypocalcemia was judged as Grade 2 or higher on the CTCAE v5.0 grading scale.

## 4 Discussion

The present study provides important real-world findings on the recently approved drug, romosozumab, in Japanese patients with osteoporosis. Significant gains were observed for lumbar, total hip, and femoral neck BMD following a 12-month treatment regimen along with significant changes in bone turnover markers. The drug was generally well tolerated. Patients switching from other osteoporotic drugs, especially denosumab, displayed lower gains in lumbar and total hip BMD, while co-administration of a vitamin D3 analogue might prevent hypocalcemia.

Clinical trials, such as the FRAME study (Cosman et al., 2016) and Ishibashi's study (Ishibashi et al., 2017) have shown the efficacy of romosozumab. However, there are key distinctions to be made between clinical trial data and real-world data. The first is the rigorous selection and exclusion criteria specific to clinical trials. In the FRAME study and Ishibashi's study, patients with a T-score of −3.5 or lower, prior osteoporosis drug use, secondary osteoporosis, 25OHD of 20 ng/mL or less, and age over 90 years were excluded. However, in actual clinical situations, many such patients consult their physician for medication. Therefore, observational research plays an important role to bridge the gap between clinical trials and the real world. For example, unlike data obtained overseas, approximately 90% of the Japanese suffer from a vitamin D deficiency or insufficiency as a complication of osteoporosis (Holick et al., 2012; Tamaki et al., 2017). In real-world practice, our approach in osteoporosis treatment is prompt therapeutic intervention without waiting for vitamin D improvement to a sufficient level. It was reported that the presence or absence of vitamin D supplementation during the use of bisphosphonates did not significantly affect a BMD increase (Nakamura et al., 2017). Furthermore, Ebina et al. found romosozumab to significantly increase BMD even in a state of vitamin D deficiency/insufficiency (Ebina et al., 2020). Therefore, the treatment of osteoporosis with vitamin D deficiency is an opportunity to produce valuable data, and accumulating evidence on the administration and follow-up of romosozumab treatment is clinically important, especially in Japan. In addition, no previous studies have shown the effectiveness of romosozumab for secondary osteoporosis. We observed that romosozumab was effective in both secondary and in primary osteoporosis. In other clinical trials under strict conditions, romosozumab was used after a washout period in the absence of treatment so as to investigate the uninfluenced benefits of romosozumab itself. However, in actual osteoporosis treatment, there are also many switch cases in which the effects of previous osteoporosis treatment are insufficient or the drug has been discontinued due to unexpected side effects; such situations may require the use of romosozumab despite residual effects from the prior treatment. Therefore, it is very meaningful to examine the clinical effect of romosozumab without setting a washout period, in line with actual clinical practice. In this study, romosozumab produced significant increases in BMD as compared with baseline at both 6 and 12 months, regardless of the pretreatment drug. However, in switch cases from such bone resorption inhibitors as bisphosphonates and denosumab, the rate of increase in BMD was significantly lower than in the treatment-naïve group. Bisphosphonates are believed to suppress bone formation by lining cells (Gasser et al., 2000), which may persist after switching to romosozumab. In fact, the baseline value of P1NP in the bisphosphonate group was lower than that in the treatment-naïve and teriparatide groups. Therefore, it was considered that the increase in BMD was hampered due to delayed action of bone formation mechanisms. In the denosumab group, the rate of BMD increase might have been smaller compared with the treatment-naïve group due to a rebound increase in remodeling following discontinuation. We presumed that the increase rate of BMD was relatively smaller than that in the naïve group because TRACP-5b, a major bone resorption marker, increased due to denosumab discontinuation, thereby leading to the activation of osteoclasts and progression of bone resorption, which sustained the effect of denosumab. This may be the reason why the bone resorption marker was increased only in the denosumab group. As was also witnessed in the STRUCTURE study, romosozumab treatment after a switch from bisphosphonates produced a significant elevation in BMD at the lumbar spine and total hip in our study (Langdahl et al., 2017). Furthermore, extension of the phase II romosozumab study has revealed successful treatment with romosozumab after denosumab (Kendler et al., 2019). According to the study by Kendler et al., treatment with denosumab followed by romosozumab produced no significant increase in BMD at the total hip, which was different from our study. It could be that the study included subjects with relatively mild symptoms (average baseline T-scores at the total hip and femoral neck were −1.48 and −1.83, respectively), younger subjects (average age was 65 years), and patients who had received romosozumab for over 2 years prior to denosumab. On the other hand, our study contained subjects with lower baseline BMD (average baseline T-scores at the total hip and femoral neck were −2.51 and −3.01, respectively). It was therefore possible that the significant improvement of BMD in our study was attributable to the lower T-scores being more susceptible to the effects of romosozumab. Moreover, the administration of romosozumab is strictly limited to 12 months or less for therapeutic use in Japan, and so the patients naïve to romosozumab were thought to be more responsive to the drug. The above factors may explain the differing outcomes in our study to other results. Overall, however, this investigation demonstrates that romosozumab remains effective regardless of the presence or type of prior osteoporosis regimen and without a specified washout time.

In the FRAME study, the change rate of serum-corrected calcium levels peaked at 2 weeks after drug administration, and the decrease rate was approximately −3.0% (Cosman et al., 2016). This rate in our study was largest at −3.7% at 12 months. We also investigated the rate of change in serum-corrected calcium after administration of romosozumab according to renal function, with no remarkable findings noted. However, the change rate was lower in subjects with co-administration of a vitamin D3 analogue than in those without. Therefore, supplementation may prevent hypocalcemia by combined use with romosozumab.

Regarding serious adverse events, we witnessed a cardiovascular event of cerebellar infarction. The patient could be discharged from a rehabilitation facility with no major sequelae, and a causal relationship with romosozumab is still unknown. Romosozumab produced no remarkable differences in the incidence of cardiovascular events compared with a placebo group in the FRAME study; however, in the ARCH study, the incidence of cardiovascular events was significantly higher than that in an alendronate administration group (Cosman et al., 2016; Saag et al., 2017). Therefore, clinicians should be vigilant during the administration of romosozumab to patients at high risk of cardiovascular events.

As limitations of this study, the following factors require further consideration: 1) not all patients were in a state of sufficient vitamin D or calcium, 2) for patients with a history of previous osteoporosis medication, no washout period of the previous drug was set; there was a possibility that the previous drug affected the results, 3) as the observation period of this study was short at 1 year, longer follow-up for adverse events and new fractures is needed, and 4) to investigate the relationship between a risk of cardiovascular events and romosozumab, studies involving more subjects are required. Lastly, since this study mainly focused on changes in BMD, it lacked the statistical power to evaluate the antifracture efficacy of romosozumab in all of the subgroups. In clinical practice, BMD improvement is a valuable indicator of osteoporosis treatment.

## 5 Conclusions

In conclusion, romosozumab is now attracting considerable attention as a new therapeutic drug for osteoporosis with its dual effect of promoting bone formation and suppressing bone resorption. In this study, romosozumab showed good therapeutic effects on BMD and was generally well tolerated in a real-world setting, regardless of osteoporosis type or treatment history. More clinical data are needed to evaluate its efficacy with existing therapeutic agents, optimal treatment conditions, and the sequential therapies following its use.

## Transparency document

Transparency document. Image 1

## CRediT Authorship contribution Statement

TK: Data Curation, Methodology, Writing - Original Draft, Visualization.

TS: Investigation, Methodology.

MN: Investigation, Data Curation, Methodology.

MS: Data Curation, Formal Analysis.

AM: Investigation.

JT: Investigation, Supervision.

YN: Conceptualization, Investigation, Writing – Review & Editing, Project Administration, Supervision.

## Funding

This research did not receive any specific grant from funding agencies in the public, commercial, or not-for-profit sectors.

## Declaration of competing interest

The authors declare no competing interests.

## Appendix A Supplementary data

Supplementary material Image 2
