# Supplementary material for: Real-world effects and adverse events of romosozumab in Japanese osteoporotic patients: A prospective cohort study
Source: Bone Rep. 2021 Apr 16;14:101068. doi: 10.1016/j.bonr.2021.101068 (PMC8085670; doi:10.1016/j.bonr.2021.101068)
Supplement: Supplementary file 2 — Supplementary material [file mmc2.pptx]

## Slide 1
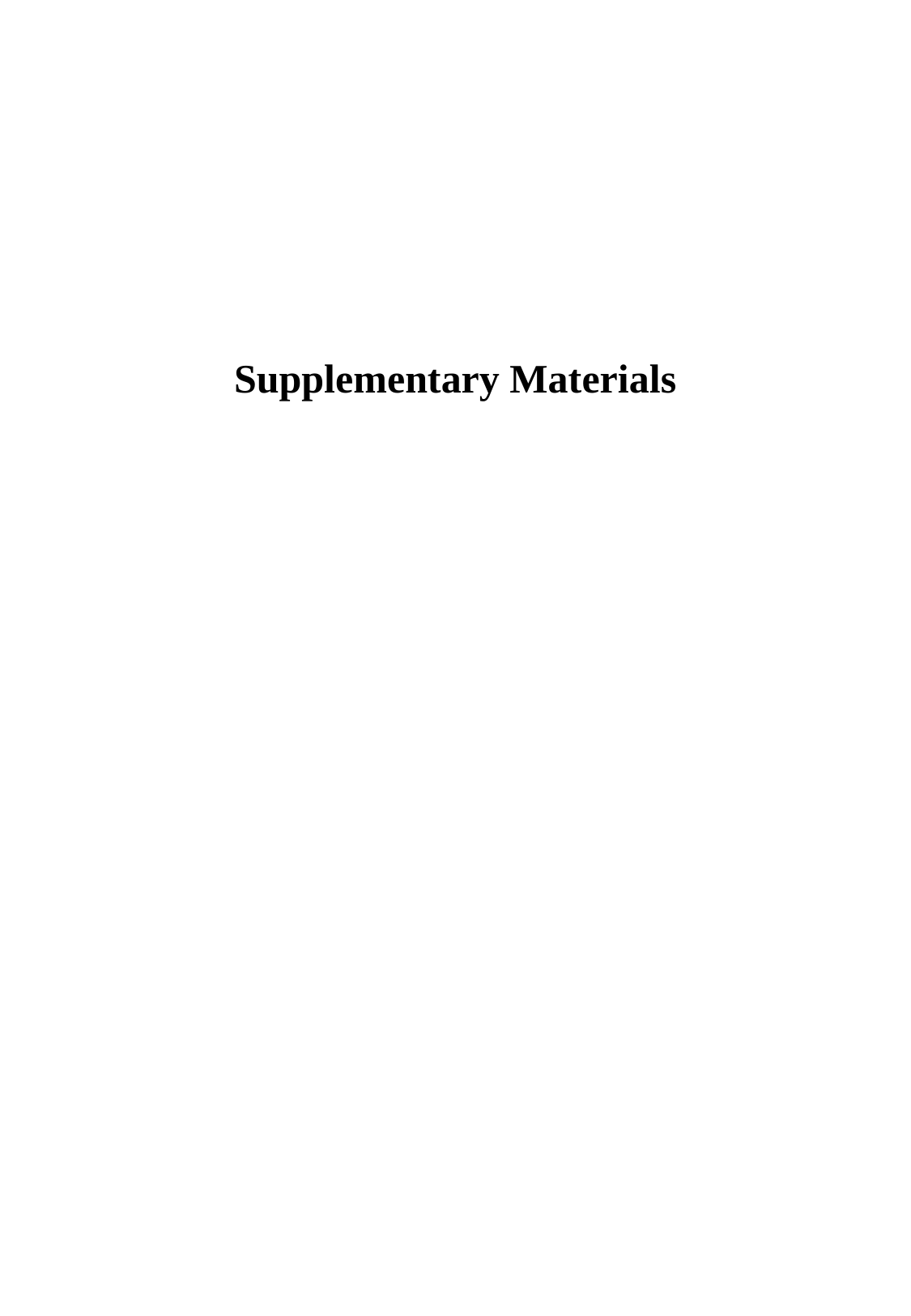

Supplementary Materials

## Slide 2
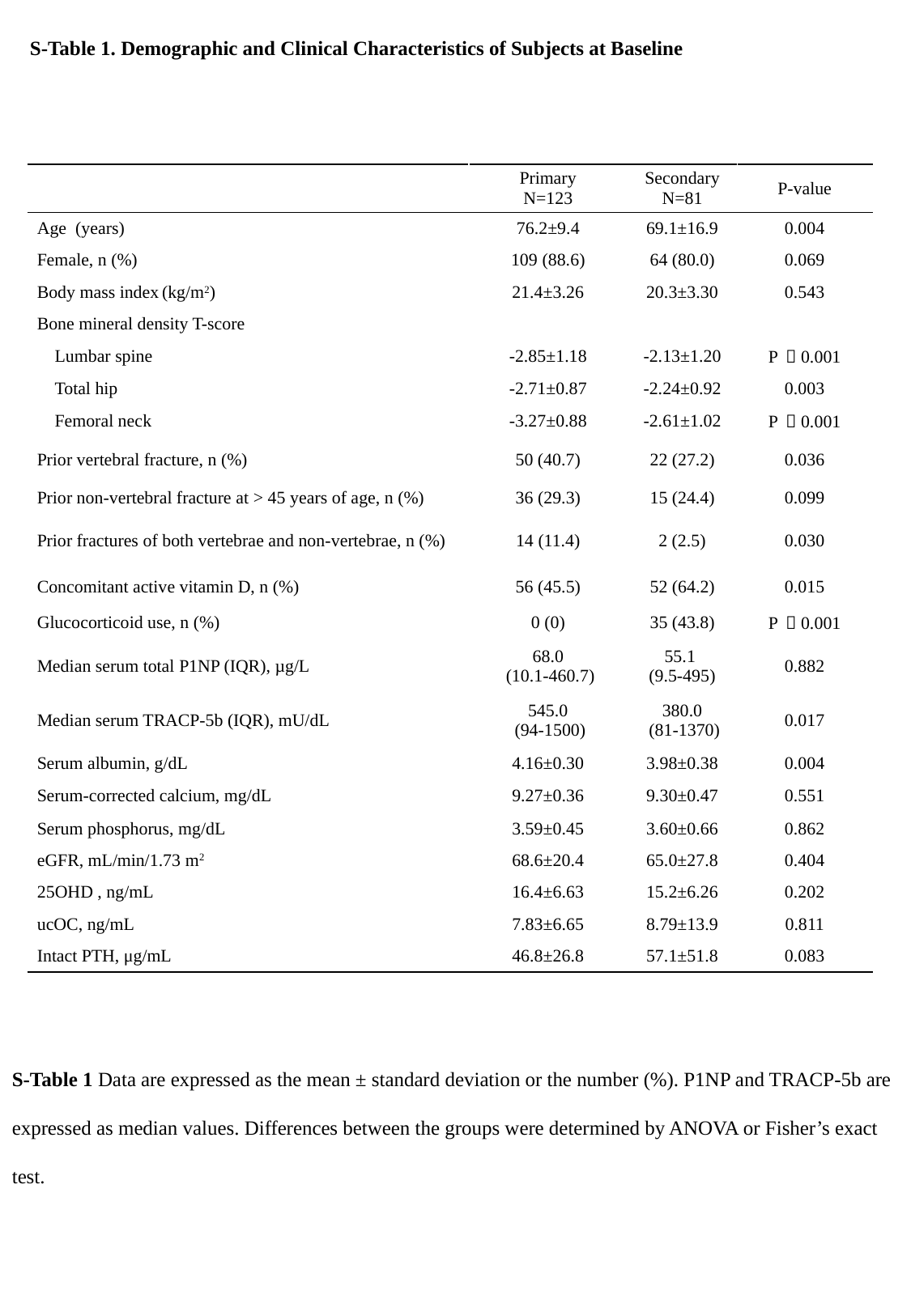

S-Table 1. Demographic and Clinical Characteristics of Subjects at Baseline
| | | | |
| --- | --- | --- | --- |
| | Primary N=123 | Secondary N=81 | P-value |
| Age (years) | 76.2±9.4 | 69.1±16.9 | 0.004 |
| Female, n (%) | 109 (88.6) | 64 (80.0) | 0.069 |
| Body mass index (kg/m2) | 21.4±3.26 | 20.3±3.30 | 0.543 |
| Bone mineral density T-score | | | |
| Lumbar spine | -2.85±1.18 | -2.13±1.20 | P ＜0.001 |
| Total hip | -2.71±0.87 | -2.24±0.92 | 0.003 |
| Femoral neck | -3.27±0.88 | -2.61±1.02 | P ＜0.001 |
| Prior vertebral fracture, n (%) | 50 (40.7) | 22 (27.2) | 0.036 |
| Prior non-vertebral fracture at > 45 years of age, n (%) | 36 (29.3) | 15 (24.4) | 0.099 |
| Prior fractures of both vertebrae and non-vertebrae, n (%) | 14 (11.4) | 2 (2.5) | 0.030 |
| Concomitant active vitamin D, n (%) | 56 (45.5) | 52 (64.2) | 0.015 |
| Glucocorticoid use, n (%) | 0 (0) | 35 (43.8) | P ＜0.001 |
| Median serum total P1NP (IQR), µg/L | 68.0 (10.1-460.7) | 55.1 (9.5-495) | 0.882 |
| Median serum TRACP-5b (IQR), mU/dL | 545.0 (94-1500) | 380.0 (81-1370) | 0.017 |
| Serum albumin, g/dL | 4.16±0.30 | 3.98±0.38 | 0.004 |
| Serum-corrected calcium, mg/dL | 9.27±0.36 | 9.30±0.47 | 0.551 |
| Serum phosphorus, mg/dL | 3.59±0.45 | 3.60±0.66 | 0.862 |
| eGFR, mL/min/1.73 m2 | 68.6±20.4 | 65.0±27.8 | 0.404 |
| 25OHD , ng/mL | 16.4±6.63 | 15.2±6.26 | 0.202 |
| ucOC, ng/mL | 7.83±6.65 | 8.79±13.9 | 0.811 |
| Intact PTH, μg/mL | 46.8±26.8 | 57.1±51.8 | 0.083 |
S-Table 1 Data are expressed as the mean ± standard deviation or the number (%). P1NP and TRACP-5b are expressed as median values. Differences between the groups were determined by ANOVA or Fisher’s exact test.

## Slide 3
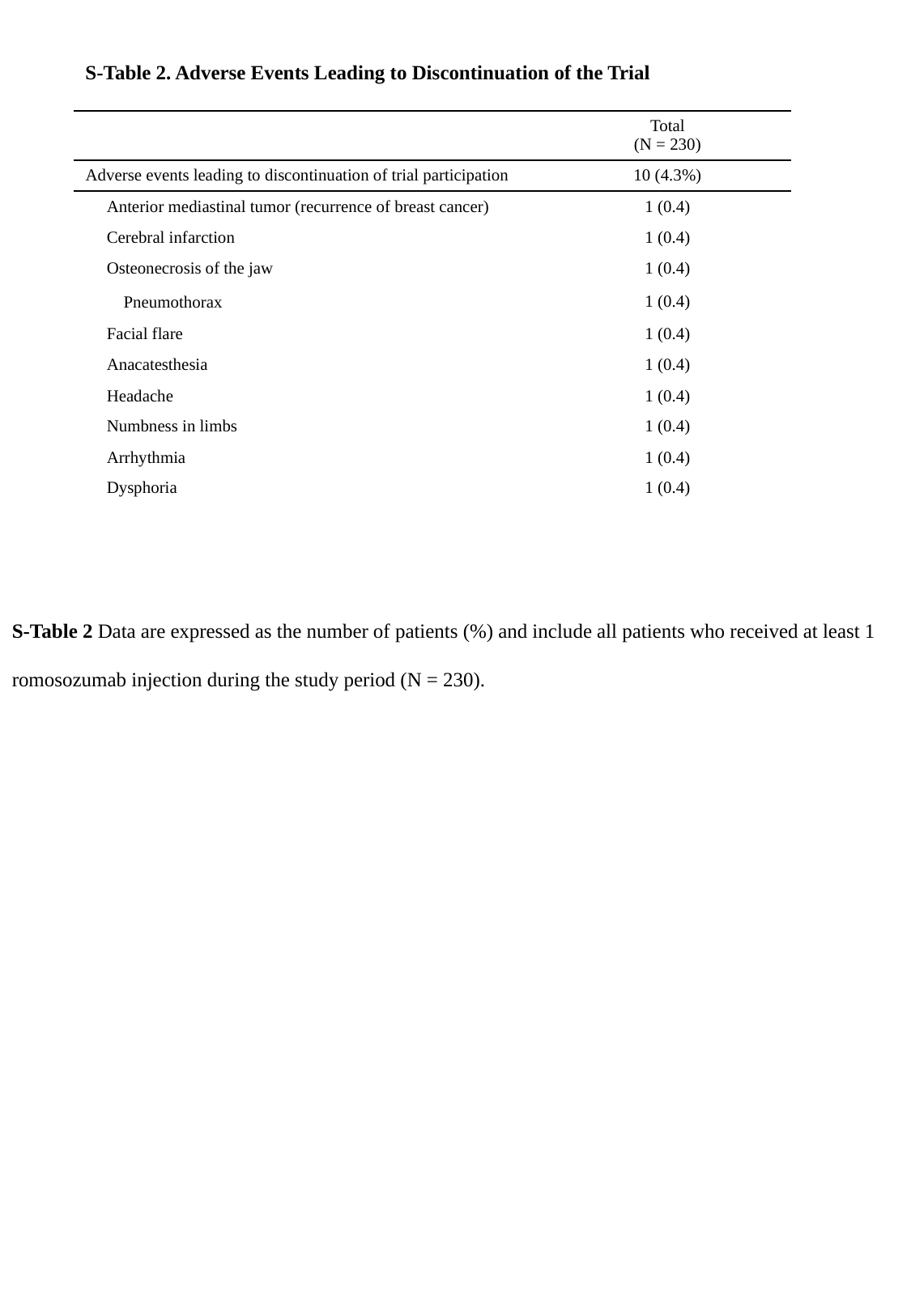

S-Table 2. Adverse Events Leading to Discontinuation of the Trial
| | Total (N = 230) |
| --- | --- |
| Adverse events leading to discontinuation of trial participation | 10 (4.3%) |
| Anterior mediastinal tumor (recurrence of breast cancer) | 1 (0.4) |
| Cerebral infarction | 1 (0.4) |
| Osteonecrosis of the jaw | 1 (0.4) |
| Pneumothorax | 1 (0.4) |
| Facial flare | 1 (0.4) |
| Anacatesthesia | 1 (0.4) |
| Headache | 1 (0.4) |
| Numbness in limbs | 1 (0.4) |
| Arrhythmia | 1 (0.4) |
| Dysphoria | 1 (0.4) |
S-Table 2 Data are expressed as the number of patients (%) and include all patients who received at least 1 romosozumab injection during the study period (N = 230).

## Slide 4
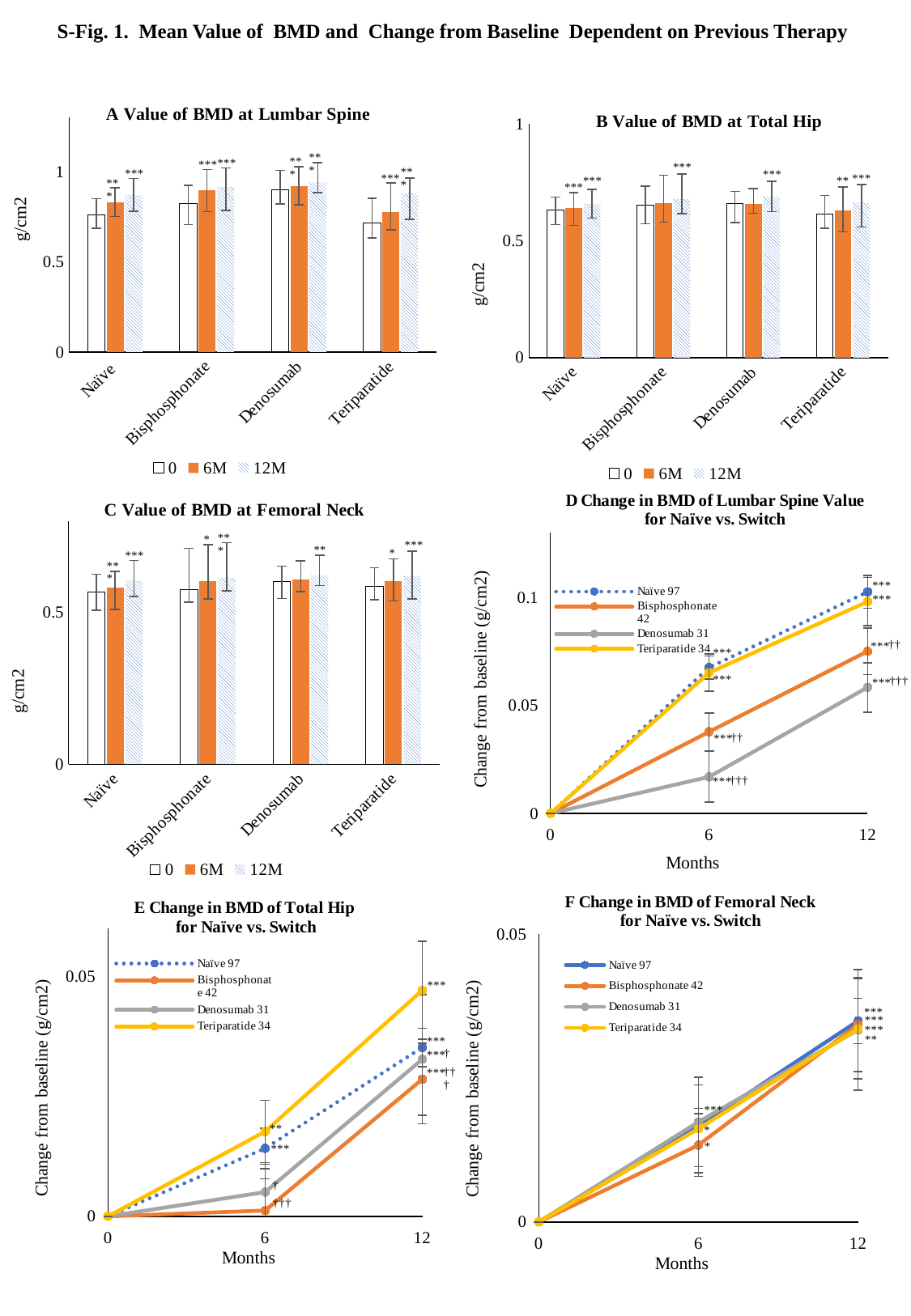

S-Fig. 1. Mean Value of BMD and Change from Baseline Dependent on Previous Therapy
### Chart
| Category | 0 | 6M | 12M |
|---|---|---|---|
| Naïve | 0.758 | 0.8314999999999999 | 0.869 |
| Bisphosphonate | 0.821 | 0.896 | 0.915 |
| Denosumab | 0.899 | 0.918 | 0.9395 |
| Teriparatide | 0.717 | 0.775 | 0.88 |
### Chart
| Category | 0 | 6M | 12M |
|---|---|---|---|
| Naïve | 0.6305000000000001 | 0.6415 | 0.6575 |
| Bisphosphonate | 0.6515 | 0.6615 | 0.679 |
| Denosumab | 0.659 | 0.657 | 0.689 |
| Teriparatide | 0.6134999999999999 | 0.63 | 0.66425 |
### Chart
| Category | Naïve 97 | Bisphosphonate 42 | Denosumab 31 | Teriparatide 34 |
|---|---|---|---|---|
| 0 | 0.0 | 0.0 | 0.0 | 0.0 |
| 6 | 0.06756250000000001 | 0.03778947368421051 | 0.016896551724137933 | 0.06516875000000003 |
| 12 | 0.10259278350515465 | 0.07507692307692308 | 0.058366666666666664 | 0.09806060606060611 |
### Chart
| Category | 0 | 6M | 12M |
|---|---|---|---|
| Naïve | 0.566 | 0.583 | 0.6045 |
| Bisphosphonate | 0.575 | 0.60425 | 0.6135 |
| Denosumab | 0.6025 | 0.609 | 0.623 |
| Teriparatide | 0.5847499999999999 | 0.602 | 0.62 |
### Chart
| Category | Naïve 97 | Bisphosphonate 42 | Denosumab 31 | Teriparatide 34 |
|---|---|---|---|---|
| 0 | 0.0 | 0.0 | 0.0 | 0.0 |
| 6 | 0.014166666666666668 | 0.001194871794871797 | 0.005035714285714278 | 0.017656250000000012 |
| 12 | 0.03517708333333334 | 0.028565000000000007 | 0.032727586206896545 | 0.047090909090909086 |
### Chart
| Category | Naïve 97 | Bisphosphonate 42 | Denosumab 31 | Teriparatide 34 |
|---|---|---|---|---|
| 0 | 0.0 | 0.0 | 0.0 | 0.0 |
| 6 | 0.01665263157894737 | 0.013346153846153848 | 0.017357142857142842 | 0.016175000000000012 |
| 12 | 0.03490104166666667 | 0.034274999999999986 | 0.03336206896551723 | 0.03356451612903227 |

## Slide 5
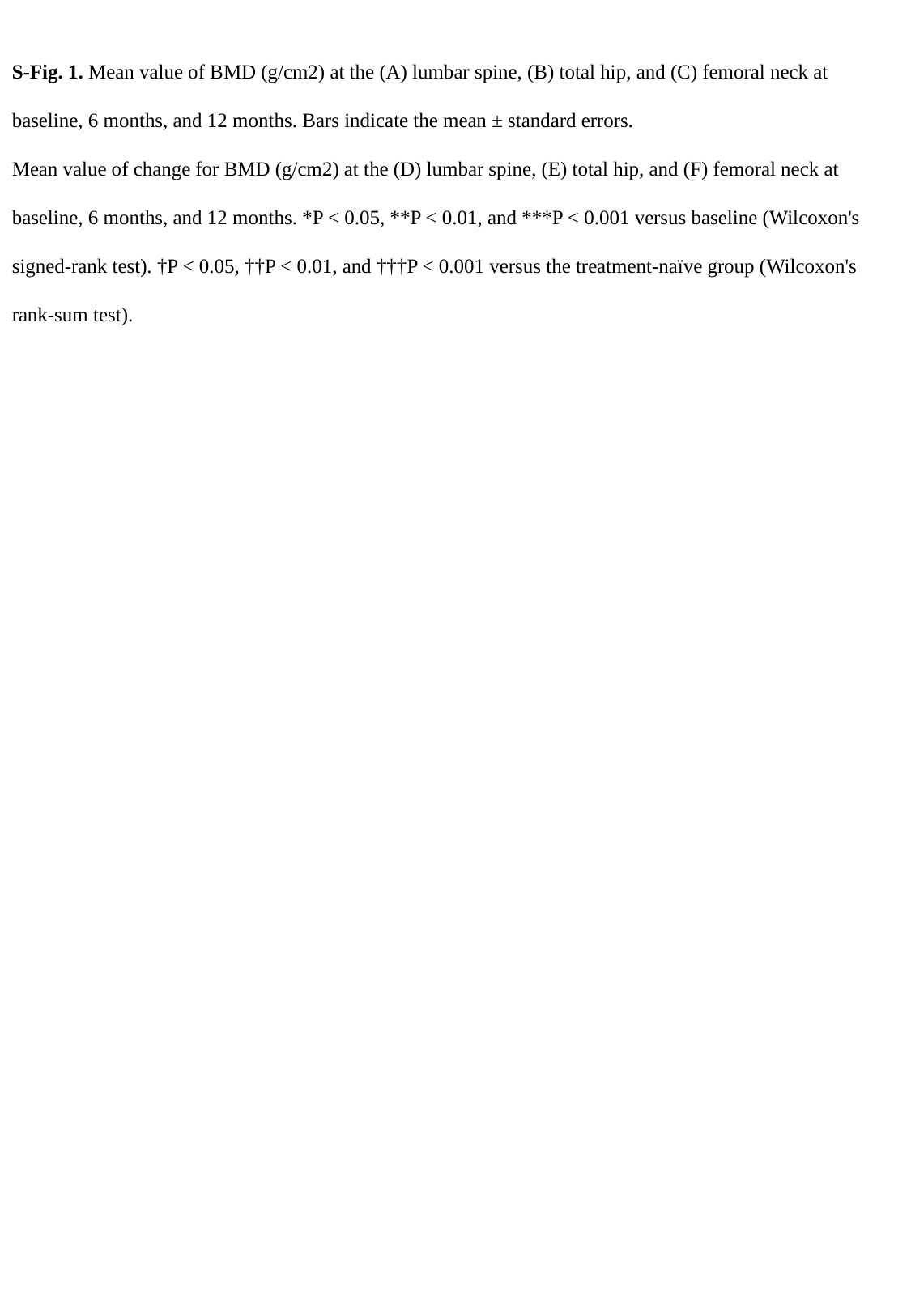

S-Fig. 1. Mean value of BMD (g/cm2) at the (A) lumbar spine, (B) total hip, and (C) femoral neck at baseline, 6 months, and 12 months. Bars indicate the mean ± standard errors.
Mean value of change for BMD (g/cm2) at the (D) lumbar spine, (E) total hip, and (F) femoral neck at baseline, 6 months, and 12 months. *P < 0.05, **P < 0.01, and ***P < 0.001 versus baseline (Wilcoxon's signed-rank test). †P < 0.05, ††P < 0.01, and †††P < 0.001 versus the treatment-naïve group (Wilcoxon's rank-sum test).

## Slide 6
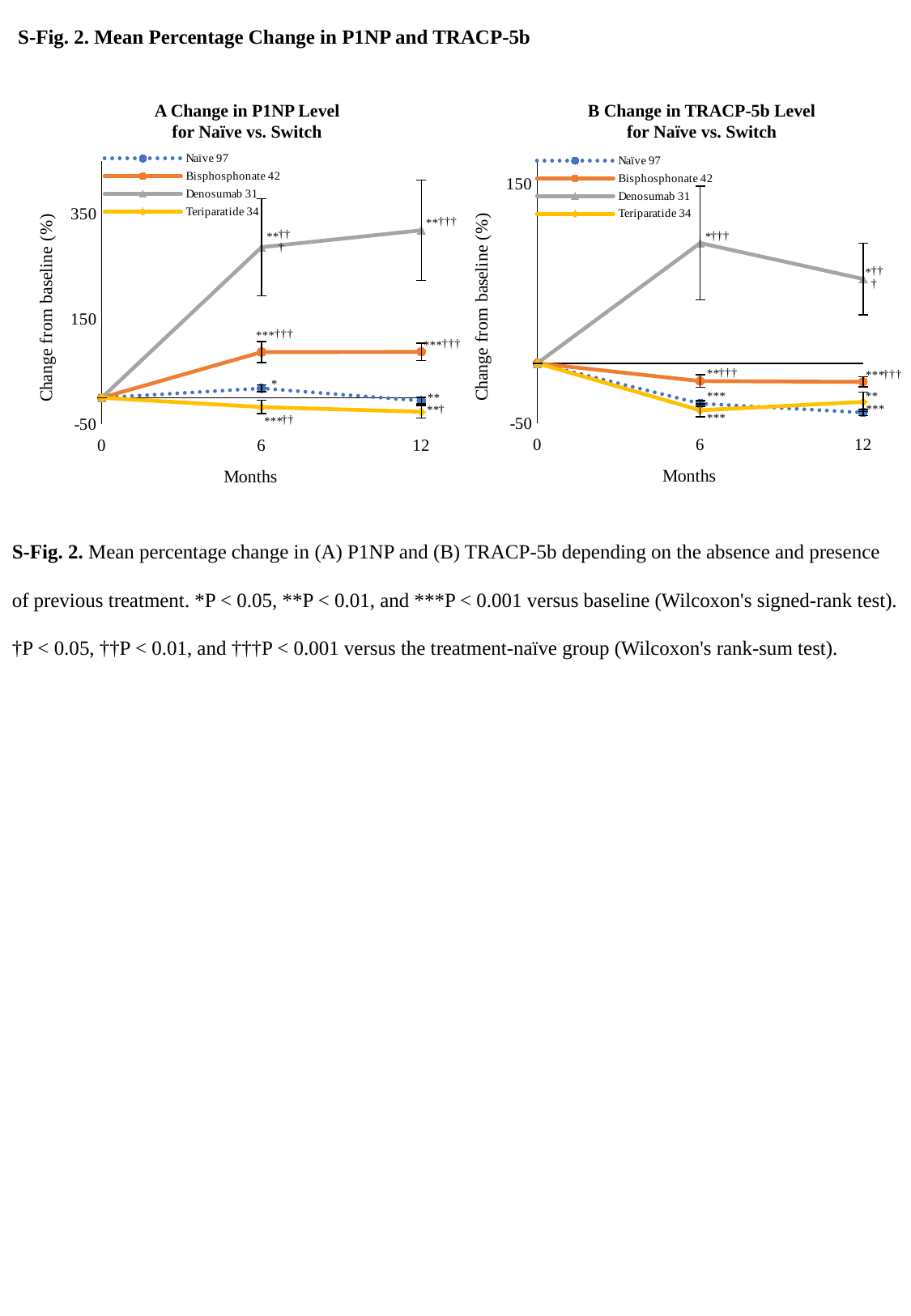

S-Fig. 2. Mean Percentage Change in P1NP and TRACP-5b
A Change in P1NP Level
for Naïve vs. Switch
B Change in TRACP-5b Level
for Naïve vs. Switch
### Chart
| Category | Naïve 97 | Bisphosphonate 42 | Denosumab 31 | Teriparatide 34 |
|---|---|---|---|---|
| 0 | 0.0 | 0.0 | 0.0 | 0.0 |
| 6 | -33.7 | -14.9 | 100.5 | -39.3 |
| 12 | -41.2 | -15.6 | 70.5 | -32.3 |
### Chart
| Category | Naïve 97 | Bisphosphonate 42 | Denosumab 31 | Teriparatide 34 |
|---|---|---|---|---|
| 0 | 0.0 | 0.0 | 0.0 | 0.0 |
| 6 | 17.9 | 86.7 | 286.1 | -17.7 |
| 12 | -5.7 | 87.1 | 318.2 | -26.6 |S-Fig. 2. Mean percentage change in (A) P1NP and (B) TRACP-5b depending on the absence and presence of previous treatment. *P < 0.05, **P < 0.01, and ***P < 0.001 versus baseline (Wilcoxon's signed-rank test). †P < 0.05, ††P < 0.01, and †††P < 0.001 versus the treatment-naïve group (Wilcoxon's rank-sum test).

## Slide 7
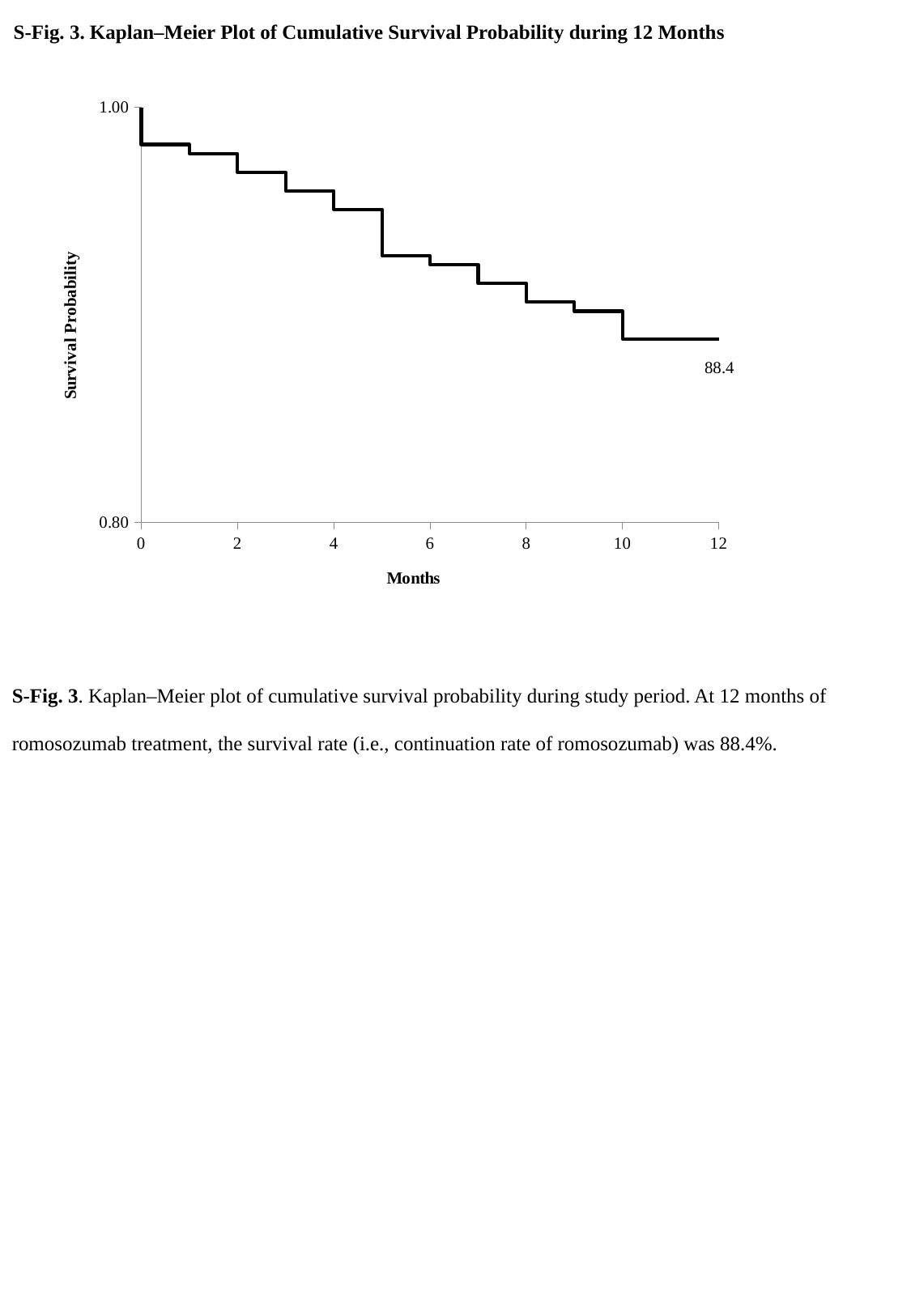

S-Fig. 3. Kaplan–Meier Plot of Cumulative Survival Probability during 12 Months
### Chart
| Category | 系列1 |
|---|---|88.4
S-Fig. 3. Kaplan–Meier plot of cumulative survival probability during study period. At 12 months of romosozumab treatment, the survival rate (i.e., continuation rate of romosozumab) was 88.4%.
